# Effects of closed-loop automatic control of the inspiratory fraction of oxygen (FiO_2_-C) on outcome of extremely preterm infants – study protocol of a randomized controlled parallel group multicenter trial for safety and efficacy

**DOI:** 10.1186/s12887-019-1735-9

**Published:** 2019-10-21

**Authors:** Christian A. Maiwald, Hendrik J. Niemarkt, Christian F. Poets, Michael S. Urschitz, Jochem König, Helmut Hummler, Dirk Bassler, Corinna Engel, Axel R. Franz, FiO2-C Study Group, Axel R. Franz, Corinna Engel, Christian F. Poets, Helmut Hummler, Michael S. Urschitz, Jochem König, Hendrik J. Niemarkt, Dirk Bassler, Christian A. Maiwald, Gabriele von Oldershausen, Iris Bergmann, Monika Weiss, Caroline J. B. R. Wichera, Andreas Eichhorn, Michael Raubuch, Birgit Schuler, Mark Schoberer, Sonja Trepels-Kottek, Thomas M. K. Völkl, Sibylle C. Horsinka, Edmondo N. L. Hammond, Christoph von Buch, Norbert Teig, Susanne Dettmers, Hans Thorsten Körner, Birte Tröger, Annika Ander, Axel Hübler, Barbara Seipolt, Lars Mense, Thomas Hoehn, Klaus Lohmeier, Hans-Jörg Bittrich, Kathrin Roefke, Christian von Schnakenburg, Klaus Niethammer, Hans Fuchs, Daniel Klotz, Monika Wolf, Sarah Kabisch, Anna Koluch, Sandra Idel, Bettina Bohnhorst, Corinna Peter, Sascha Meyer, Harald Sauer, Kathrin Lorenz, Joachim Kühr, Sandra Holz, Ulrich H. Thome, Benjamin W. Ackermann, Corinna Gebauer, Catrice Celanowski, Hubert Fahnenstich, Cecil Kannan, Eva Mildenberger, André Kidszun, Julia Winter, Rolf F. Maier, Hana Voss, Ralf Pallacks, Kirsten M. Lang, André Gatti, Andreas W. Flemmer, Susanne Herber-Jonat, Marcus Krüger, Daniela Reber, Julia Sandkötter, Katja Masjosthusmann, Florian Urlichs, Thomas Frank, Michael Schroth, Christian Grillhösl, Jochen Kittel, Holger Michel, Sven Wellmann, Hans-Christoph Schneider, Anja Mayer, Hans-Martin Lode, Daniel Lorenz, Axel T. Bosk, Torben Lindner, Matthias Vochem, Patrick Neuberger, Jörg Arand, Marc R. Mendler, Jochen Essers, Christian Bender, Jessica Beckmann, Ralf Rauch, Ulrich Bernbeck, Kirsten Glaser, Johannes Wirbelauer, André A. Kroon, Tom Goos, Thilo Mohns, Henrica L. M. van Straaten, Estelle E. M. Mulder, Mara Hesse

**Affiliations:** 1 Department of Neonatology, University Children’s Hospital Tübingen, Calwerstr. 7, 72076 Tübingen, Germany; 2 Center for Pediatric Clinical Studies (CPCS), University Children’s Hospital Tübingen, Calwerstr. 7, 72076 Tübingen, Germany; 3 Máxima Medical Centre Veldhoven, Veldhoven, The Netherlands; 4 Institute of Medical Biostatistics, Epidemiology and Informatics, Medical Center of the Johannes Gutenberg-University Mainz, Mainz, Germany; 5 Children’s Hospital University of Ulm, Ulm, Germany; 6 UniversitätsSpital Zürich, Zürich, Switzerland

**Keywords:** Oxygen, Closed-loop automated control of the inspiratory fraction of oxygen (FiO_2_-C), Infant, premature, Intermittent hypoxemia and hyperoxemia

## Abstract

**Background:**

Most extremely low gestational age neonates (ELGANS, postmenstrual age at birth (PMA) < 28 completed weeks) require supplemental oxygen and experience frequent intermittent hypoxemic and hyperoxemic episodes. Hypoxemic episodes and exposure to inadequately high concentrations of oxygen are associated with an increased risk of retinopathy of prematurity (ROP), chronic lung disease of prematurity (BPD), necrotizing enterocolitis (NEC), neurodevelopmental impairment (NDI), and death beyond 36 weeks PMA.

Closed-loop automated control of the inspiratory fraction of oxygen (FiO_2_-C) reduces time outside the hemoglobin oxygen saturation (SpO_2_) target range, number and duration of hypo- and hyperoxemic episodes and caregivers’ workload. Effects on clinically important outcomes in ELGANs such as ROP, BPD, NEC, NDI and mortality have not yet been studied.

**Methods:**

An outcome-assessor-blinded, randomized controlled, parallel-group trial was designed and powered to study the effect of FiO_2_-C (in addition to routine manual control (RMC) of FiO_2_), compared to RMC only, on death and severe complications related to hypoxemia and/or hyperoxemia. 2340 ELGANS with a GA of 23 + 0/7 to 27 + 6/7 weeks will be recruited in approximately 75 European tertiary care neonatal centers. Study participants are randomly assigned to RMC (control-group) or FiO_2_-C in addition to RMC (intervention-group). Central randomization is stratified for center, gender and PMA at birth (< 26 weeks and ≥ 26 weeks).

FiO_2_-C is provided by commercially available and CE-marked ventilators with an FiO_2_-C algorithm intended for use in newborn infants. The primary outcome variable (composite of death, severe ROP, BPD or NEC) is assessed at 36 weeks PMA (or, in case of ROP, until complete vascularization of the retina, respectively). The co-primary outcome variable (composite outcome of death, language/cognitive delay, motor impairment, severe visual impairment or hearing impairment) is assessed at 24 months corrected age.

**Discussion:**

Short-term studies on FiO_2_-C showed improved time ELGANs spent within their assigned SpO_2_ target range, but effects of FiO_2_-C on clinical outcomes are yet unknown and will be addressed in the FiO_2_-C trial. This will ensure an appropriate assessment of safety and efficacy before FiO_2_-C may be implemented as standard therapy.

**Trial registration:**

The study is registered at www.ClinicalTrials.gov: NCT03168516, May 30, 2017.

## Background

Approximately 0.5% of all neonates (i.e., about 25,000 infants per year in Europe) are extremely low gestational age neonates (ELGANs), i.e. have a gestational age at birth (GA) < 28 completed weeks. The vast majority of ELGANs requires supplemental oxygen in addition to positive pressure respiratory support and frequently experience intermittent hypoxemic and hyperoxemic episodes. Intermittent hypoxemic episodes are predominantly caused by recurrent apnea due to immature development of the respiratory neuronal network (recently reviewed [1, 2]) but also secondary to active exhalation during mechanical ventilation [3]. Hyperoxemic episodes are usually a consequence of inappropriate adjustments of FiO_2_ (during routine manual control of FiO_2_ (RMC) but potentially also during closed-loop automated control of FiO_2_ (FiO_2_-C)).

### Complications of prematurity associated with recurrent hypoxemic episodes

#### Retinopathy of prematurity (ROP)

Observational data indicated that both, severe and prolonged hypoxemic episodes [4–6], and wide fluctuations in oxygen levels [7], increase the risk of ROP. Whereas a better control of SpO_2_-levels was associated with a decreased risk of ROP [8].

#### Death and neurodevelopmental impairment (NDI)

Observational studies (recently reviewed in [9]) as well as SpO_2_ data recorded during the Canadian Oxygen Trial (COT [10],) suggest that late deaths (i.e. deaths beyond 36 weeks postmenstrual age (PMA)) and NDI (both cognitive and particularly motor impairment) are linked to hypoxemic episodes, particularly those of more than 60s duration [6].

#### Necrotizing enterocolitis (NEC)

The NeOProM (Neonatal Oxygen Prospective Meta-analysis) collaboration reported a lower rate of severe NEC (defined as NEC leading to abdominal surgery or death) in infants assigned to the higher SpO_2_ target range (91–95% compared to 85–89%) [11], which was linked to a lower proportion of time spent with SpO_2_ < 80%.

### Complications of prematurity associated with hyperoxemic episodes

Considering that breathing room air (i.e., FiO_2_ = 0.21) leads to a relative hyperoxia compared to intrauterine oxygen partial pressures (PO_2_) and oxidative stress in preterm infants, hyperoxia, caused by inadequately high FiO_2_, is likely associated with long-term adverse effects [12].

#### ROP

The causal relationship between prolonged inappropriate exposure to high oxygen concentrations and ROP has long been established [13, 14]. More recently, the NeOProM studies showed increased rates of ROP with the higher SpO_2_-target range (91–95%) [11]. Finally, implementation of the higher SpO_2_-target range based on the results of the NeOProM studies, was associated with an increase in ROP rates in a recent observational study [15].

#### Death and NDI

Data from experimental studies in rodents indicate that higher levels of oxygen (e.g., FiO_2_ 0.80 for 2 to 24 h [16–18]) trigger apoptotic neurodegeneration or white matter damage in the brain. These effects have been reviewed by Back et al. [19].

#### Chronic lung disease of prematurity (BPD)

Hyperoxia enhancing generation of reactive oxygen species triggers inflammatory processes, tissue damage and cell death in the preterm infant’s lung, eventually resulting in an increased risk of BPD development (recently reviewed in [20]).

### Controlling of FiO_2_

To protect ELGANs from the detrimental effects of hypoxemic and hyperoxemic episodes, it can be assumed that PO_2_ (and in appropriate simplification SpO_2_) must be kept within a narrow target range. To achieve this goal despite the infants’ irregular breathing patterns and variations in lung aeration and function, frequent cautious adjustments of FiO_2_ are required, which are challenging, time consuming and often impossible due to limited personnel resources.

It has repeatedly been shown that FiO_2_-C increases the time infants spent within the SpO_2_-target range and reduces the burden of hyper−/hypoxemia while being safe and accurate in short-term studies (reviewed in [21, 22]). The effects of FiO_2_-C on clinically relevant outcomes measures (such as the hypoxia and hyperoxia-associated complications of prematurity described above) and the safety of its long-term continuous application, however, have yet to be elucidated.

## Methods/design

### Trial objectives

The proposed trial was designed and is powered to compare the effect of FiO_2_-C in addition to manual adjustments, in comparison with RMC of FiO_2_ only, on death, NDI and severe complications of prematurity thought to be related to hypoxia/hyperoxia in ELGANs.

### Trial design

This is an outcome-assessor-blinded, randomized-controlled, multicenter parallel group comparison of phase III for superiority (evaluating FiO_2_-C in addition to RMC of FiO_2_ in comparison to RMC of FiO_2_ only) in ELGANs.

In Germany, this study is also considered as a phase IV pharmaceutical trial on safety of the investigational medication ‘oxygen’ using different modes of administration (decision of the German authority BfArM according to §4 para. 23,1 of the German Pharmaceutical Act). This may not apply to other countries.

### Setting

Patients will be recruited in approximately 75 European tertiary care neonatal centers. Recruitment has started in Germany and is intended to expand to additional sites in other European countries, after appropriate approvals will have been obtained.

### Patients

#### Inclusion criteria

- GA at birth 23 + 0/7 to 27 + 6/7 weeks

#### Exclusion criteria

- Decision not to provide full life support / decision for palliative care only before study entry
- Severe congenital abnormalities (particularly those affecting respiratory, cardiovascular or gastrointestinal function or long-term neuro-cognitive development, whereas patent ductus arteriosus, patent foramen ovale (PFO), and atrial septal defects type II (ASDII) are not considered a congenital anomaly in preterm infants)
- Postnatal age > 48 h
- Lack of parental consent
- Lack of device enabling closed-loop automatic control of FiO_2_ before randomization

### Randomization and allocation concealment

Study participants are randomly assigned in a 1:1 ratio to FiO_2_-C in addition to RMC of FiO_2_ (test intervention) or RMC of FiO_2_ only (control intervention).

A web-based randomization tool provided by the Interdisciplinary Center of Clinical Studies at the University Medical Center of the Johannes Gutenberg University Mainz is being used in this study. This program enables bound (into the same treatment group) or free (into different treatment groups) randomization of multiples based on parental choice and the number of available devices enabling FiO_2_-C.

A minimization algorithm is applied to preferentially aim for an even distribution of treatment assignment in both GA strata (i.e. < 26 weeks and ≥ 26 weeks; 1st priority) and both gender strata (2nd priority) within each center.

### Blinding

This study is outcome-assessor-blinded, meaning that the personnel performing the ophthalmological examinations throughout the initial hospitalization as well as the personnel performing the neurocognitive evaluation at 24 months corrected age will be blinded to the infants’ treatment group assignment. Blinding of doctors, nurses, and parents is not possible with this type of study interventions.

### Study intervention

FiO_2_-C is provided by commercially available and CE marked infant ventilators with a FiO_2_-C algorithm intended for use in preterm infants. The FiO_2_-C algorithm must have been tested in human infants and shown to increase the %-time spent in the assigned SpO_2_ target range or to reduce the time in hypoxemia or hyperoxemia or to reduce the incidence/duration of hypoxemic or hyperoxemic episodes.

Each FiO_2_-C algorithm should be applied in its “optimal mode” (with respect to potentially variable settings provided by the manufacturer such as: averaging time of SpO_2_-input, response/wait-time, etc.) based on either evidence in the literature or consensus of the users.

Manual adjustments are encouraged whenever automatic FiO_2_ settings seem sub-optimal. In case of FiO_2_/SpO_2_-oscillations brought about by FiO_2_-C, settings need to be adapted or FiO_2_-C has to be temporarily interrupted.

Whenever possible, every study center will use only one type of FiO_2_-C algorithm.

Infants in the control group are treated (whenever possible) with the same type of infant ventilator for respiratory support (FiO_2_-C turned off) and RMC of FiO_2_ is applied by bedside nurse and medical staff throughout the initial hospitalization.

Care is taken in both groups that all staff are informed about the relevance of intermittent hypoxemia and hyperoxemia and trained to execute prudent and careful RMC of FiO_2_. This training may include a standard operating procedure for RMC, where the speed of increase/decrease in FiO_2_ depends on the magnitude of deviation from the SpO_2_ target range as previously described [23, 24].

The intervention should start as soon as possible after randomization and within 48 h after birth. The scheduled end of the study intervention is any of the following (whichever comes first):

- death
- discharge home from hospital
- transfer to another hospital where FiO_2_-C is not available (whereas such transfer is discouraged)
- a PMA of 36 + 0/7 weeks
- *final* discontinuation of positive pressure respiratory support, which does not include limited periods without positive pressure support for weaning.
*If the infant requires positive pressure respiratory support again for any reason, the infant should again be supported by FiO*_*2*_ *-C (provided a device supporting FiO*_*2*_ *-C is available) until (other) criteria for scheduled end of study intervention are met.*
- a PMA of > 32 + 0/7 weeks provided the following two additional criteria of respiratory stability are both met:
- ◦ A) FiO_2_ = 0.21 for ≥48 h (for this criterion limited time periods with higher FiO_2_ for rescue or for recovery of intermittent hypoxemia will not be considered)

and

- ◦ B) less than 5 intermittent hypoxemic episodes with an SpO_2_ < 80% per 8 h shift.

*If criteria A) or B) are no longer met, the infant should again be supported by the FiO*_*2*_ *-C device until (other) criteria for scheduled end of study intervention are met.*

- a PMA of > 32 + 0/7 weeks if the infant has to be transferred to an intermediate care unit where FiO_2_-C is not available

*If the infant is re-admitted to intensive care, the infant should again be supported by FiO*_*2*_ *-C (provided a device supporting FiO*_*2*_ *-C is available) until (other) criteria for scheduled end of study intervention are met.*

After the end of the study intervention, all study participants will be treated according to the state of the art care and local standards without further requirements or restrictions.

### Concomitant interventions and medication

Any concomitant medication that is clinically considered necessary for the patient will be allowed within the study, except for the control group, where closed-loop automatic control of FiO_2_ or any other automatic control of airway pressure/respiratory support etc. based on SpO_2_ or other vital signals are not allowed.

### SpO_2_ measurements to guide FiO_2_-C

All FiO_2_-Controllers should be based on SpO_2_-data generated by the same pulse oximeter technology (Masimo). In general, pre-ductal SpO_2_-sensor placement is preferred to guide FiO_2_-C as long as echocardiography demonstrates a patent ductus arteriosus.

### SpO_2_-targets and alarm settings

The SpO_2_-target range selected by a center for clinical routine has to fulfill the following criteria:

- Study centers need to have a written guideline on SpO_2_-target range to ensure that the same SpO_2_-target range is applied in both study groups
- The SpO_2_-target has to be within the range of 87–95% (may include 87% and/or 95%),
- Care has to be taken that the same SpO_2_-target ranges are applied in clinical routine and in both study groups

### Documentation of the study intervention

In both study groups, the type of respiratory support, the type of ventilator and the application of FiO_2_-C have to be documented daily during the intervention period on a treatment log.

### Primary outcome

The primary outcome measure is a composite of death, BPD or NEC assessed at 36 weeks PMA and severe ROP assessed when full vascularization of the retina is documented.

### Definitions of components of the primary outcome

#### Severe ROP

Defined as any ROP stage 3 or higher, or acute posterior ROP, or any ROP in Zone 1, or any treatment for ROP. ROP will be diagnosed at routine ophthalmological examinations, beginning at a PMA of 32 weeks according to international recommendations and local standards until complete vascularization of the retina [25]. The severity of ROP will be graded according to the international classification [26].

#### BPD

Defined as requiring positive pressure support or supplemental oxygen at 36 weeks ±2 days PMA, including an oxygen reduction test for infants requiring less than 0.3 FiO_2_, representing ‘moderate’ or ‘severe’ BPD according to the National Institute of Child Health and Development consensus definition [27].

#### NEC

Defined as modified Bell stage ≥IIa [28] until 36 weeks PMA.

### Co-primary outcome

The co-primary outcome (tested in a hierarchical design) is the composite outcome of death, language or cognitive delay, motor impairment, severe visual impairment or hearing impairment, all assessed at 24 ± 1 months corrected age.

### Definitions of components of the co-primary outcome

*Language or Cognitive delay:* Defined as a language or cognitive composite score at the Bayley Scales of Infant Development 3rd edition [29] of < 85.

*Motor impairment:* Defined as a Gross Motor Function Classification System (GMFCS) score of 2–5 [25].

*Severe visual impairment:* Defined as best corrected vision in the better eye yields a visual acuity less than 6/60 m (20/200 ft) according to the relevant doctor’s reports / discharge summary.

*Severe hearing impairment:* need for a hearing aid or cochlear implant.

Any clinical suspicion of previously undiagnosed visual or hearing problems during the FiO_2_-C follow-up visit requires a referral to an eye specialist or an pedaudiologist.

*If the parents refuse the assessment at the study center or if Bayley test cannot be performed:*

Other assessments of neurocognitive and motor development will betaken into account, if parents refuse to attend the follow-up.

Cognitive- and language-composite-scores will then be imputed as follows:

A score “> 85” will be imputed if

- a different cognitive test has been performed elsewhere and scored higher than 1SD below the mean
- the family pediatrician/doctor/health professional caring for the child or the parents rate the infant as “normal”

A score “< 85” will be imputed if

- a different cognitive test has been performed elsewhere and scored lower than 1SD below the mean
- the family pediatrician/doctor/health professional caring for the child or the parents rate the infant as “delayed” or “impaired”.

Any such imputation will be described in the final report and the scientific publication.

### Secondary outcomes

Key secondary outcome variables are the individual components of the primary (death, severe ROP, BPD, NEC) and co-primary outcome variables (death, cognitive delay or language delay, motor impairment (GMFCS score of 2–5 [30]), as well as severe visual or hearing impairment,, the composite scores of the Bayley Scales (3rd edition), the rate of cerebral palsy (CP) according to the criteria defined by the European network ‘Surveillance of CP in Europe’, and the GMFCS score.

In addition to ‘severe ROP’ as component of the primary outcome, the ‘ROP Severity Score’ (also entitled ‘ROP activity and structure score’) [31] is assessed as secondary outcome, enabling better differentiation and likely being more relevant for functional outcome.

### Ethical considerations

The Helsinki Declaration shall be applied to the clinical trial, as well as Good Clinical Practice (GCP). The protocol was submitted and approved by the Ethics Committee of the University Hospital Tübingen as the lead ethics committee. Furthermore, the relevant ethics committees responsible for any of the participating study sites will have to approve participation of the site.

### Community engagement

A freely accessible web page for *FiO*_*2*_*-C* has been set up (www.fioc-study.eu), providing an overview of aims, partners, study outline, progress and milestones, meetings, findings and news.

### Form of consent

Written informed consent from parents or legal guardians is required for participation in the study.

### Insurance

Where required by national law, insurance will be obtained for all study patients.

### Sample size, power and study duration

The required sample size was calculated for the primary research hypothesis that the implementation of FiO_2_-C reduces the cumulative incidence of the composite primary outcome (death, severe ROP, BPD, or NEC).

The co-primary research hypothesis is that FiO_2_-C also reduces death or severe NDI (see outcome measures for details). These hypotheses are assessed as a-priori ordered hypotheses, where the co-primary hypothesis will only be tested in a confirmatory manner if the primary hypothesis has been confirmed. Consequently, no correction for multiple testing will be performed.

We assume that,

- the cumulative incidence of the primary composite outcome of this study is 50% in the control group
- FiO_2_-C reduces the burden of severe hypoxemia/hyperoxemia by 25–50% and (based on the assumption that (again) 25–50% of the outcome is associated with recurrent hypo−/hyperoxemia) effects a relative risk reduction in this outcome by at least 12.5%.

In summary, we assume a reduction in the primary outcome from 50% (in the control group) to 44% in the intervention group (FiO_2_-C).

**Fig. 1:**
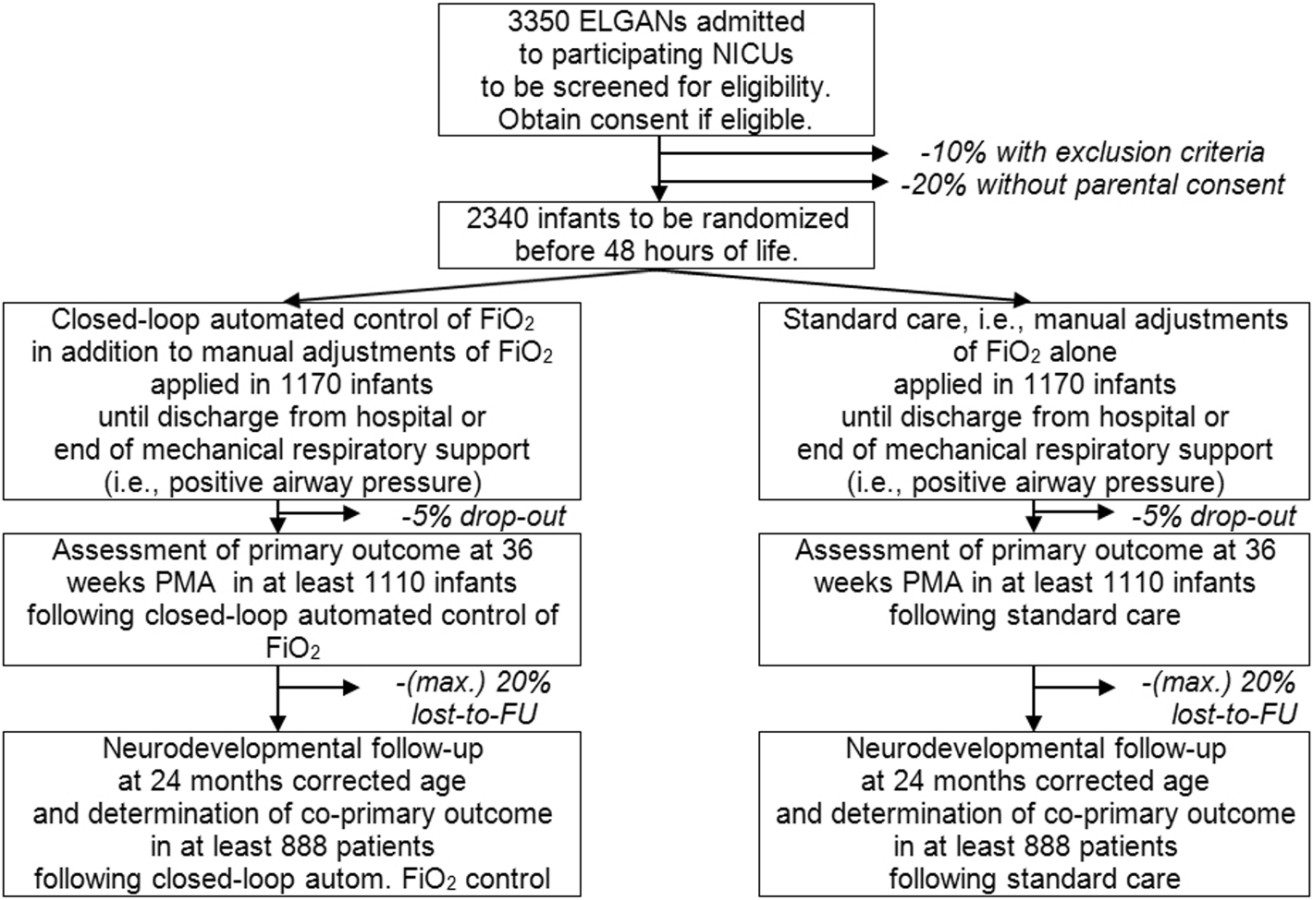
Anticipated Trial Flow

Sample size calculations were based on a Χ^2^-test, assuming a power of 80% and a significance level of 5%. Based on these assumptions, 1110 infants are required in each treatment group (total 2220 infants). Because all components of this primary outcome will be determined during the initial hospitalization (i.e. until first discharge from neonatal care), the rate of drop-out before ascertainment of the primary outcome will be low as < 5%. Hence, a total of 2340 infants need to be enrolled and randomized (see Fig. 1).

Assuming an incidence of 50% for the co-primary outcome in the control group and a relative risk reduction (RRR) of 25% for the co-primary outcome in the FiO_2_-C group, the proposed sample size will have a power > 80% to prove this difference even if up to 20% of randomized infants will be lost to follow-up until 24 months corrected age.

It is estimated that about 90% of all ELGANs will qualify for inclusion into this study without any exclusion criteria. Estimating a participation rate of 80%, approximately 3350 infants have to be screened.

We estimate a recruitment of about 65 patients per month and therefore the recruitment phase of the study will last for approximately 36 months. The individual participation in the study will be about 27 months (between 56 and 91 days of treatment – depending on GA at birth - with an additional follow up to 24 months corrected age).

### Data analysis

Analysis of the primary outcome will be based on the intention to treat analysis set, which comprises all randomized patients. Portions of infants with primary endpoint will be compared in a statistical model that accounts for the factors considered by the randomization procedure and the randomization of twins and other multiples. The treatment effect will be reported as a risk ratio and as a risk difference with 95% confidence interval. The co-primary outcome will be assessed only if superiority of FiO_2_-C with respect to the primary outcome is confirmed at the 2-sided level of 0.05. This hierarchical testing procedure maintains a multiple type I error of 0.05. All statistical analyses will be described in detail in a statistical analysis plan completed before closure of the database. An interim analysis for efficacy is not intended.

### Monitoring safety

An independent Data Monitoring Committee (DMC) is instituted and monitors recruitment, compliance, and safety parameters after 50, 100, 200 and 300 patients have completed 44 + 0/7 weeks PMA, and after every 200 patients have reached this age thereafter.

### Safety parameters

Safety parameters monitored by the DMC include:

Early deaths (for the DMC defined as < 44 weeks PMA), late deaths (for the DMC defined as ≥44 weeks PMA), all deaths, BPD, discharge on home oxygen or home positive pressure respiratory support, severe ROP, NEC, (focal) intestinal perforation requiring laparotomy, PDA requiring treatment, intraventricular hemorrhage >grade 2, cystic periventricular leukomalacia. Because the safety parameters include components of the primary outcome, the incidence rates and 95% confidence intervals, these parameters will be ‘coded’ as “safety parameter A-I”.

Furthermore, safety analyses include occurrence and rates of reported adverse events and incidents by treatment group.

### Regulatory aspects

#### Trial sponsor

Sponsor of the FiO_2_-C-trial is the University Hospital Tübingen, Geissweg 3, 72,076 Tübingen, Germany. Contact is available at fioc@med.uni-tuebingen.de.

#### Medical ethics committees

At the time of submission, the relevant ethics committee in Germany approved the study. Applications for approvals are currently underway in additional countries (e.g., the Netherlands and Switzerland).

#### National Regulatory/competent authorities

At the time of submission, the National Regulatory/Competent Authority of Germany (BfArM) approved the study. Authority approval may not be necessary elsewhere – but this will be determined in collaboration with the relevant ethics committees.

## Discussion

### Need for a trial

Oxygen is one of the drugs most frequently used in ELGANs and yet, our knowledge on the optimal level of oxygen in arterial blood (or in appropriate simplification the optimal target range for SpO_2_) and even optimal technology for monitoring oxygen levels is incomplete [32–34]. Short-term studies in preterm infants demonstrated that FiO_2_-C improved the time within the assigned SpO_2_ target range. In these studies, percent time within the assigned SpO_2_ target range increased by approximately 10% points to around 70–90% and the improvement was independent of the SpO_2_ target range, the FiO_2_-C algorithm, and the proportion of time spent within SpO_2_ target range in the control-group [21, 22, 35, 36]. It is, however, unclear if more time spent within the assigned SpO_2_ target range will also translate into positive long-lasting effects on clinically relevant outcomes. For example, despite higher proportions of time spent within the SpO_2_ target range, FiO_2_-C might on the one hand side reduce the amplitude of SpO_2_ fluctuations, but, at the same time increase the frequency of SpO_2_ oscillations and thereby might carry additional risks. This randomized controlled trial will ensure an appropriate assessment of safety and efficacy of FiO_2_-C, before it is implemented into standard care.

### Discussion of the study intervention period

The study intervention period was chosen because Di Fiore et al. showed that hypoxemic episodes evolve over the first 2 weeks of life and hence starting the intervention within 48 h after birth seems appropriate. This will enable a reasonable time frame to inform parents, even if birth of the infant occurs at night or on weekends, and to enable a meaningful parental decision on participation.

As described by Di Fiore et al. [5] and confirmed by Poets et al. [6], hypoxemic episodes occurring beyond the 4th week of life are more strongly associated with adverse long-term outcomes than hypoxemic episodes occurring within the first 4 weeks of life. Hence, the study intervention should not end at 32 weeks PMA. Infants with prolonged and frequent hypoxemic episodes beyond this age may benefit most from effective FiO_2_-C.

### Discussion of chosen population

Because diseases thought to be related to inappropriate use of oxygen such as ROP and BPD essentially only occur in ELGANs, an assessment of efficacy and safety of long-term application of FiO_2_-C can only be performed in this patient population.

### Discussion of chosen SpO_2_-target range

The NeOProM collaboration has shown that the higher SpO_2_-target range of 91 to 95% is associated with a decreased risk of early deaths at 18 to 24 months corrected age and NEC, but with an increased risk of ROP [11]. Furthermore, a post-hoc analysis of the BOOST-II data indicated that a higher proportion of time within the assigned target range could enhance this beneficial effect [37]. Consequently, in the FiO_2_-C trial the lower limit of the center-specific SpO_2_ target range has to be set to ≥87% SpO_2_.

## Trial status

Protocol version 4: April, 26th, 2018. Recruitment has started in July 2018 and is expected to be finalized in July 2021. The last patient out (after follow-up) will be expected in October 2023.

## Data Availability

Data sharing is not applicable to this article as no datasets were generated or analyzed for the current manuscript.

## Abbreviations

ASDII: Atrial Septal Defect II
BfArM - Federal Institute for Drugs and Medical Devices: Bundesinstitut für Arzneimittel und Medizinprodukte
BMBF - German Federal Ministry of Education and Research: Bundesministerium für Bildung und Forschung
BPD: Chronic lung disease of prematurity
CP: Cerebral palsy
eCRF: Electronical case report form
ELGANs: Extremely low gestational age neonates
FiO_2_-Controller / FiO_2_-C: Closed loop automated control of FiO_2_
GA: Gestational age
GMFCS: Gross Motor Function Classification System
NDI: Neurodevelopmental impairment
NEC: Necrotizing enterocolitis
paO_2_: Arterial oxygen partial pressures
PFO: Patent foramen ovale
PO_2_: Oxygen partial pressures
RMC: Routine manual control
ROP: Retinopathy of prematurity
RRR: Relative risk reduction
